# Small sample sizes in the study of ontogenetic allometry; implications for palaeobiology

**DOI:** 10.7717/peerj.818

**Published:** 2015-03-10

**Authors:** Caleb Marshall Brown, Matthew J. Vavrek

**Affiliations:** 1 Royal Tyrrell Museum of Palaeontology, Drumheller, Alberta, Canada; 2 Department of Natural History, Royal Ontario Museum, Toronto, Ontario, Canada

**Keywords:** Size, Shape, Sample size, Fossil, Error, Growth, *Alligator*, Morphometrics, Isometry, Palaeontology

## Abstract

Quantitative morphometric analyses, particularly ontogenetic allometry, are common methods used in quantifying shape, and changes therein, in both extinct and extant organisms. Due to incompleteness and the potential for restricted sample sizes in the fossil record, palaeobiological analyses of allometry may encounter higher rates of error. Differences in sample size between fossil and extant studies and any resulting effects on allometric analyses have not been thoroughly investigated, and a logical lower threshold to sample size is not clear. Here we show that studies based on fossil datasets have smaller sample sizes than those based on extant taxa. A similar pattern between vertebrates and invertebrates indicates this is not a problem unique to either group, but common to both. We investigate the relationship between sample size, ontogenetic allometric relationship and statistical power using an empirical dataset of skull measurements of modern *Alligator mississippiensis*. Across a variety of subsampling techniques, used to simulate different taphonomic and/or sampling effects, smaller sample sizes gave less reliable and more variable results, often with the result that allometric relationships will go undetected due to Type II error (failure to reject the null hypothesis). This may result in a false impression of fewer instances of positive/negative allometric growth in fossils compared to living organisms. These limitations are not restricted to fossil data and are equally applicable to allometric analyses of rare extant taxa. No mathematically derived minimum sample size for ontogenetic allometric studies is found; rather results of isometry (but not necessarily allometry) should not be viewed with confidence at small sample sizes.

## Introduction

Morphometric analyses are becoming increasingly common in biological studies to quantify and investigate biological shape (Adams, Rohlf & Slice, 2013; Bookstein, 1985; Bookstein, 1997; Fabre et al., 2013; Klingenberg, 1996; Klingenberg, 1998; Mitteroecker et al., 2013; Rohlf, 1990; Samuels, Meachen & Sakai, 2013; Zelditch, Sheets & Fink, 2003; Zelditch et al., 2004). One of the key uses of morphometric methods in both neontology and palaeobiology is as a more objective and repeatable means to quantify patterns of shape change and size change within organisms. Scaling (allometry), often described as ‘relative growth’ or ‘differential growth,’ is the variation in shape associated with variation in size (Cock, 1966; Klingenberg, 1996), and has been an integral aspect of the study of growth in the contexts of ontogeny and evolution since Julian Huxley and George Simpson (Blackstone, 1987; Cock, 1966; Gould, 1966; Hofman, 1988; Huxley, 1932; Huxley & Teissier, 1936; Simpson, 1944; Simpson, Roe & Lewontin, 1960; Strauss, 1987). Within the context of biological scaling, traits growing (whether ontogenetically or evolutionarily) at the same rate represent isometry, whereas traits increasing at different rates represent allometry—either negative or positive relative to the reference trait. Given the nature of the data available, and the evolutionary questions of interest, much of the theoretical underpinning of scaling and allometry has been of particular interest to palaeobiologists, particularly during the ‘palaeobiological revolution’ (Alberch et al., 1979; Gould, 1966; Gould, 1977; Sepkoski & Ruse, 2008). Allometry can be generally regarded as having three levels or scales: static allometry—individual variation within an age class of a population; ontogenetic allometry—variation in a taxon as a result of a growth trajectory; and evolutionary allometry—variation between taxa due to evolutionary differences (Klingenberg, 1996). This paper concentrates on ontogenetic allometry.

Neontological studies often use scaling to elucidate relative growth of anatomical structures, patterns of polymorphism, and to differentiate between closely related taxa (Dodson, 1975a; Dodson, 1975b; Dodson, 1978; Dodson, 1979; Zelditch & Fink, 1995; Zelditch, Sheets & Fink, 2003; Zelditch et al., 2004). Similar questions are asked by palaeobiologists, but researchers are limited to a small subset of the data available to neontologists, most often consisting of hard tissue anatomy (Carrano, 2001; Chapman, 1990; Chapman & Brett-Surman, 1990; Chapman et al., 1981; Dececchi & Larsson, 2013; Dilkes, 2001; Dodson, 1975c; Dodson, 1990; Evans, 2007; Heathcote, 2004; Heinrich, Ruff & Weishampel, 1993; Kilbourne & Makovicky, 2010; Marugan-Lobon & Buscalioni, 2004; Reisz et al., 2005; Sadleir & Makovicky, 2008; Stayton & Ruta, 2006) but see Allen and colleagues (2013).

Of particular interest among many biologists is the pattern of intraspecific ontogenetic allometry, the ‘heterauxesis’ of Simpson (1953), the allometry of elements or structures relative to the total size of an organism (Alberch et al., 1979; Rice, 1997; Simpson, 1953; Strauss, 1987). This can, in theory but rarely in practice, be derived directly from multiple measurements of a single individual through its lifespan (longitudinal studies, e.g., Cock, 1966; Jungers & Fleagle, 1980), rather than the less desirable but more practical indirect method of estimating patterns of individual growth from samples of multiple individuals at various stages of ontogeny (cross-sectional studies, see Alberch et al., 1979; Gould, 1966 and citations therein). Palaeobiologists usually cannot, due to the nature of the fossil record, measure a single individual at differing times in its life, and usually must rely on bulk sampling of a population or taxon, in cross-sectional studies, to indirectly infer ontogenetic allometry. These ontogenetic trajectories illuminate the developmental dynamic of an organism’s life history. Anatomical elements showing strong positive allometric growth in extant species, such as horns, antlers and crests, may often be taxonomically diagnostic display structures and thought to be under sexual selection and, likewise, secondary sexual characters are often positively allometric (Geist, 1966; Geist, 1968; Simmons & Tomkins, 1996). Based on this, features which show strong positive allometric growth within extinct clades may be potential display structures, and under sexual selection (Brown, Russell & Ryan, 2009; Dodson, 1975c; Evans, 2007; Goodwin et al., 2006; Goodwin & Horner, 2004; Gould, 1973; Gould, 1974; Hone, Naish & Cuthill, 2011; Horner & Goodwin, 2009; Knell & Sampson, 2011; Padian & Horner, 2011; Sampson, Ryan & Tanke, 1997; Tomkins et al., 2010). Determining the pattern of relative growth of these structures is therefore often important for interpretation of their palaeobiological significance.

Isometry may not, however, represent the most appropriate null model when the scaling systems are locomotory or biomechanical in nature. Here models of geometric similarity may give way to models of elastic and/or static stress similarity, where the null hypothesis is not isometry (Biewener, 2005; McMahon, 1975).

As ontogenetic allometry is of interest to palaeobiologists, and can usually only be inferred based on multiple individuals preserved at various stages of ontogeny, there may be systemic methodological problems in the determination of ontogenetic trajectories (see Gould, 1966 and citations therein). Further compounding this problem, palaeobiologists are often limited to the small sample sizes that are associated with fossil taxa. Small sample size is arguably the most limiting factor in most palaeobiological studies, particularly those of vertebrates, and this is most evident in quantitative analyses such as morphometrics. The effect of small sample size in morphometric studies include reducing the number and type of analyses that can be performed, reducing the statistical and resolving power of those analyses, and increasing the probability of Type II error. This last point is of particular interest when a goal is to categorize each variable as positively allometric, negatively allometric, or (essentially) isometric. Because isometry is usually treated as the null, and small sample sizes will have reduced power, there will be large amounts of false isometry (incorrect conclusions of isometry, when allometry is correct) when small sample sizes are used. Cardini & Elton (2007), in their analysis of the effect of sample size in geometric morphometric analyses, illustrated that while estimates of mean size, standard deviation of size, and variance of shape were robust to small sample sizes, estimates of mean shape and static allometric trajectories were strongly affected by small sample sizes.

The implications and limitations of small sample sizes have been empirically tested and discussed in other aspects of palaeobiology, namely palaeoecology (Forcino, 2012; Grayson, 1978; Koch, 1987; Wolff, 1975) and diversity studies (de Caprariis, Lindemann & Collins, 1976; Grayson, 1981; Miller & Foote, 1996; Raup, 1975; Signor & Lipps, 1982), but this effect in morphometrics, particularly allometry, is less well understood (but see Cardini & Elton, 2007; Cobb & O’Higgins, 2004; Strauss & Atanassov, 2006).

Here we provide an empirical investigation of the practical limits that small sample size has on allometric analyses on extinct vertebrates, using an extensive ontogenetic series of a well-understood extant taxon, *Alligator mississippiensis*. We also perform a literature survey to quantify differences in sample size between neontological work and palaeontological work, and between work on vertebrate and invertebrate taxa.

## Materials

### Literature survey

In order to understand and document the range and distribution of sample sizes that have been used in previous allometric studies, a survey of studies performing intraspecies allometric (static and ontogenetic, but not evolutionary allometry) analyses was conducted. Studies were retrieved using the search term “allometry” in Google Scholar, and those investigating evolutionary allometry (i.e., those investigating scaling trends between species, not within species) were disregarded. In total, 542 samples (intraspecific ontogenies), were recorded from 102 studies (see Table S1). Many studies, specifically those comparing ontogenetic allometry between species, contained samples pertaining to more than one species, and in these cases the sample for each species was recorded individually. For all samples, the author and year, genus and species, sample size, and whether the data pertained to invertebrates or vertebrates, and extinct or extant taxa were recorded. This allowed for direct comparisons of the distributions of sample sizes between extinct and extant taxa, and between vertebrate and invertebrate taxa.

### Empirical dataset

In order to understand the relative effects of sample sizes on allometric studies, an empirical dataset of 23 linear skull measurements of *Alligator mississippiensis* was utilized. *A. mississippiensis* was chosen for a number of reasons. This taxon is well understood, has a large range of body sizes, and shows a prolonged period of growth—though debate is ongoing as to whether this is best characterized by indeterminate or prolonged determinate growth (Jacobsen & Kushlan, 1989; Lance, 2003; Wilkinson & Rhodes, 1997; Woodward, Horner & Farlow, 2011). This large range allows for investigations into the effect of shape as a result of size to make use of an extensive body size axis. Possibly because of these factors, large osteological collections exist in North American natural history museums that allow for ease of data collection, and modern *A. mississippiensis* has experienced an extensive history as modern analogue for testing ideas of extinct archosaur palaeobiology (Brochu, 1996; Dodson, 1975a).

The cranial dataset contains 108 specimens of *A. mississippiensis* (see Table S2). The measured sample includes specimens from hatchling (or near hatchling) sized individuals to large adults and as such represents a well-sampled size series for *A. mississippiensis*.  As a result, the dataset encompasses a remarkable range in skull sizes. The skull length of the largest specimen (ROM 51011—689 mm) is more than twenty times larger (in linear dimensions) than that of the smallest (ROM R 7966—29 mm). As such, it represents as broad a range of scale effects as will likely be encountered in any palaeontological analysis. The majority of the specimens do not include age data, and although we here investigate the effect of size, size can also be used as a proxy for age to approximate the effects of age. Although spanning the size range, our dataset does not equally sample each size class, with poorer sampling at the upper and lower size extremes. Subsampling simulations (see ‘Methods’) attempt to test the effect of this unequal sampling.

## Methods

All data analyses were carried out using the R software package (R Development Core Team, 2009) (v 2.13.0), with regressions using the package smatr (Warton et al., 2011; Warton et al., 2012) available on the CRAN website. Comparison of samples sizes used in ontogenetic allometry between fossil and modern taxa, and between vertebrate and invertebrate taxa, were performed using a two-sided Kolmogorov–Smirnov (KS) test to determine the probability that the two samples were drawn from the same distribution. This test not only accounts for differences in the relative position of the distribution (e.g., mean), but also the shape of the distribution (e.g., skewness, kurtosis).

### Measurements

**Figure 1:**
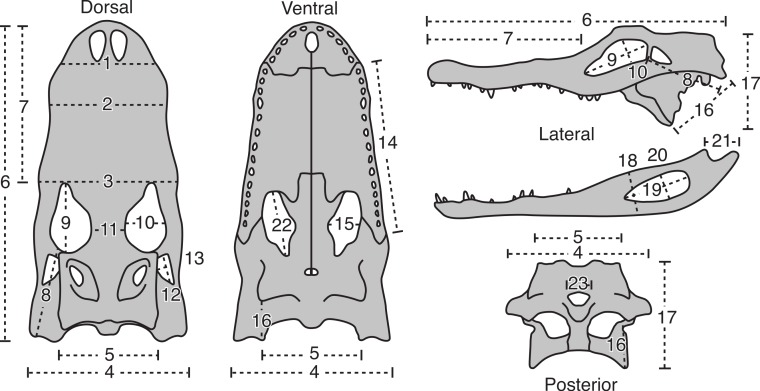
The 23 linear morphometric variables of *A. mississippiensis* skulls used in this study. For description of measurement see Table 1. Figure modified from Dodson (1975a).

Twenty-three cranial measurements were taken from each skull (see Fig. 1 and Table 1). The measurements were taken following Brown, Arbour & Jackson (2012), which were modified from Dodson (1975a). These osteological measurements represent functional complexes as opposed to dimensions of individual bones, and have biomechanical and behavioral correlates (see Dodson, 1975a). Measurements were taken from the left side, unless this side was either incomplete or damaged, in which case the right side was used. Measurements smaller than 150 mm were taken with digital calipers, and those greater than 150 mm with a fiberglass measuring tape. All measurements were taken to the nearest millimeter.

**Table 1:** Description of the 23 cranial measurements used in this study. See Fig. 1 for diagram. Modified from Dodson (1975a).

#	Description of variable
1	Skull width at posterior border of external nares
2	Skull width at 4th maxillary tooth
3	Skull width at anterior border of orbit
4	Skull width at posterior border of quadratojugals
5	Skull width across exoccipitals
6	Skull length from tip of snout to quadrates
7	Skull length from tip of snout to anterior border of orbits
8	Skull length from posterior border of orbit to external condyle of quadrate
9	Orbit length
10	Orbit width
11	Orbit separation
12	Lateral temporal fenestra length
13	Lateral temporal fenestra height
14	Maxilla length
15	Palatal fenestra width
16	Distance from the posterolateral corner of the pterygoid to the medial condyle of quadrate
17	Height of skull from pterygoid process to dorsal surface of skull, perpendicular to long axis
18	Maximum depth of jaw
19	External mandibular fenestra length
20	External mandibular fenestra width
21	Retroarticular process length, from crest of ridge posterior to articular cotyles to tip of process
22	Palatal fenestra length
23	Foramen magnum width

To assess the scale of measurement error, three skulls were repeatedly measured on twelve occasions, with at least one day between subsequent measurements periods. For this analysis of measurements error, measurements were recorded to the nearest 0.01 mm (digital calipers) and 0.1 mm (fiberglass tape). Measurement error was quantified in two ways, average deviation (the arithmetic mean of the absolute value of difference of all replicates and the replicate mean) and standard deviation. Of the 65 variables (23 for two specimens and 19 for one specimen), the average deviation ranged from 0.03 mm to 3.32 mm, with only four (4/59) caliper variables and four (4/6) tape variables above the 1.00 mm rounding threshold (Fig. S1). Neither error of caliper nor tape measurements are correlated with measurement size, but the caliper showed consistently less error (mean = 0.44 mm) than the tape (mean = 1.34 mm). Between specimens, there is no significant correlation of measurement error for each variable, indicating that certain variables are not consistently more/less likely to be prone to measurement error than others between skulls.

### Regression

The complete dataset of *Alligator mississippiensis* was analyzed for the allometric trajectories of 22 linear cranial measurements. All variables were logarithmically transformed prior to analysis. Debate concerning the utility of logarithmic transformations in allometry does exist (Mascaro et al., 2014; Packard, 2013; Smith, 1984; Sokal & Rohlf, 1995), but is not reviewed here.

Each variable (with the exception of the reference datum) was plotted against the reference datum, skull length, for the entire sample size. Basal skull length has been cited as a good reference datum for allometry studies (Gould, 1974; and references therein). Both Ordinary Least Squares (OLS) and Reduced (Standardized) Major Axis (RMA or SMA) regressions were used to determine the slopes, 95% confidence intervals of the slopes, and correlation coefficients for each variable relative to skull length. In addition to the continuous variables of slope and confidence intervals, each variable was also assigned to a categorical variable of positively allometric (95% confidence interval of slope is greater than 1), negatively allometric (95% confidence interval of slope is less than 1), or isometric (95% confidence interval of slope includes 1). The values for the slope, 95% confidence intervals of the slope, correlation coefficient, and allometric category (all based on the entire ontogenetic series—108 specimens) were recorded as the ‘true’ regression parameters for each variable, to which the subsamples were then compared.

### Subsampling

To test the effect that smaller sample sizes have on the ability to reliably obtain similar slopes and scaling categories (i.e., positive/negative allometry, isometry), the complete size series was systematically subsampled (1,000 replicates) using four distinct Monte Carlo subsample methods (Random, Even Length Binned, Even Occupancy Binned, and Adult Biased), regressed, and the results compared to the results for the entire dataset. For this study, the result for the entire sample was regarded as the ‘true’ result; see discussion below. The different subsampling techniques were employed in order to test not just the effect that sample size has on the scaling analysis, but also to test how the range and distribution of samples across the size axis affects the scaling analysis.

**Figure 2:**
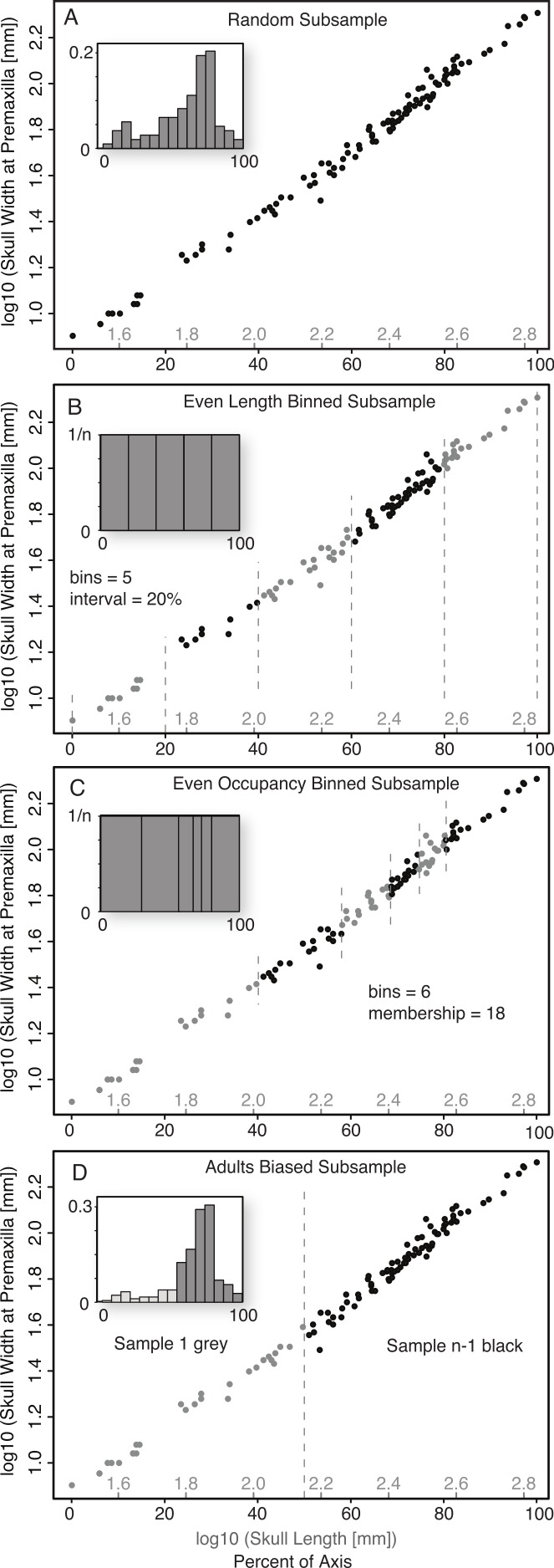
Diagram illustrating the five subsampling techniques utilized. Random Subsample (A); Even Length Binned Subsample (B); Even Occupancy Binned Subsample (C); Adults Biased (D).

*Random Subsample (without replacement).* This was the simplest form of Monte Carlo subsampling performed, and consisted of randomly selecting (without replacement) from the entire size series the number of specimens corresponding to the desired sample size, *n*. The relative position of specimens within the ontogenetic series had no influence on their probability of being selected and, other than the lack of replacement, the choice of the subsequent specimens was not affected by the choice of the preceeding specimens (Fig. 2A).

*Binned Subsamples.* Two basic methods of binned subsampling were used, occupancy-based and length-based. Both of these divided the ontogenetic series into *n* bins, with *n* being the number of samples in the replicate.

*Even Length Binned Subsample.* This method divided the size series into *n* bins, with the size of the bins determined by equal divisions of the reference variable (basal skull length)—that is, each bin represents an equal amount of the magnitude of the reference measurement (Fig. 2B). One specimen is then selected at random from each bin. This subsample method represents the best-case scenario as it both maximizes the size range (for the reference variable) of the sampled specimens, and distributes them relatively evenly across that range.

*Even Occupancy Binned Subsample.* This method divided the size series into *n* bins, with the size of the bins determined by equal occupancy of the bins—that is, each bin has the same number of specimens within it (Fig. 2C). One specimen is then selected at random from each bin. This acts to maximize the range and even out the distribution, of subsamples within the complete sample, but is dependent on the relative distribution of the sampling intensity.

*Adult Bias Subsample.* Ontogenetic datasets derived from existing samples (as opposed to captive breeding), such as museum specimens or fossil data, rarely preserve an even distribution of samples across the ontogenetic series and often show distinct biases towards sampling of large/adult specimens, and against sampling of small/juvenile specimens. To replicate this, a second method was developed to segregate all specimens into two arbitrary size classes, those with a skull length less than half that of the largest reference variable, and those with a length greater than half that of the largest reference specimen. For a given sample size (*n*), the group of larger specimens was subsampled randomly for *n* − 1 specimens, while the group of smaller specimens was subsampled for one specimen (Fig. 2D). This method simulated a sample composed largely of adults, but with one juvenile specimen (i.e., from the smaller size class).

*Subsampling intervals.* The Random subsample was performed over a range from three to 100 specimens, increasing with an interval of one specimen. ‘Even Occupancy Binned,’ and ‘Adults Bias’ subsample methods were performed over a range from 3 to 20 specimens, increasing with an interval of one specimen. The ‘Equal Length Binned’ method was performed over a range from 3 to 10 specimens. The unequal distribution of specimens across the axis of the reference specimen would not allow for smaller bin size for the non-random methods. All subsample methods were performed for 1,000 independent replicates.

### Comparison of subsamples to whole sample

Comparisons of differences in results between each subsample level, and for each variable, and the ‘true’ results allowed for determination of the sample sizes for which there are differences in the categorical scaling trends (i.e., positive allometry, isometry, negative allometry) between ‘true’ and subsample results. Any deviation of the subsample replicates from the ‘true’ results (i.e., those for all 108 specimens in the entire sample) is interpreted as error due to small sample size. For the categorical scaling trends, this was determined as the sample size at which 95% of the replicates result in the same scaling trend as that of the ‘true’ (entire) trend. For example, if the relationship of variable 1 (relative to skull length) is positively allometric for the entire sample (*n* = 108), and 93% of replicates with a sample size of 23 return the result of positive allometry, and 96% of replicates with a sample size of 24 return the result of positive allometry, the minimum sample required for variable 1 is *n* = 24. This analysis was performed for each subsample method, and the resulting minimum sample size compared between subsample methods.

There are three potential errors that can be made when concluding which scaling trend best describes the relative growth of one variable relative to another. Firstly, the two variables do grow at the same rate (i.e., their slope is not different from 1.00) and are isometric, but one concludes that they are growing at different rates or allometry (their slope is not 1.00). This represents an incorrect rejection of a true null hypothesis (isometry), and is regarded as false allometry, which is akin to Type I error. Secondly, the two variables do grow at different rates (i.e., their slope is not 1.00), and are thus allometric, but one incorrectly concludes they are isometric (their slope is not different from 1.00). This represents an incorrect failure to reject a false null hypothesis (isometry), and is regarded as false isometry, which is akin to Type II error. Finally, the two variables do grow at different rates (i.e., their slope is not 1.00), and are thus allometric, and one correctly concludes they are allometric, but the sign of the allometry is wrong (e.g., they are negatively allometric, but are found to be positively allometric). This is referred to here as ‘sign error.’

It is important to note that this study does not represent a simulation analysis (with known/set parameters), but rather subsampling of an empirical dataset. As such the allometric results of the entire sample do not represent the true pattern for an entire population/species, but are themselves only inferences from larger subsamples of an entire unsamplable population/species. For this reason, the use of ‘Type I error’ to describe false allometry, and ‘Type II error’ to describe false isometry, is not meant imply statistical definitions of these errors, but only to illustrate their general similarity to the deviations from the whole analysis.

Equations describing this relationship between minimum sample size, and slope, given the empirical data were compared using Akaike Information Criterion (AIC) to evaluate goodness of fit given model complexity.

It should be noted that the distribution of the raw dataset (as well as the log-transformed dataset) fail Shapiro–Wilk tests for normality. As a result, linear regression statistics extracted from the data may not accurately represent the pattern of scaling. Despite this limitation, we feel the patterns documented in this paper are informative, and would encourage similar analyses with larger, more robust datasets.

## Results

### Distributions of sample size in allometry

**Figure 3:**
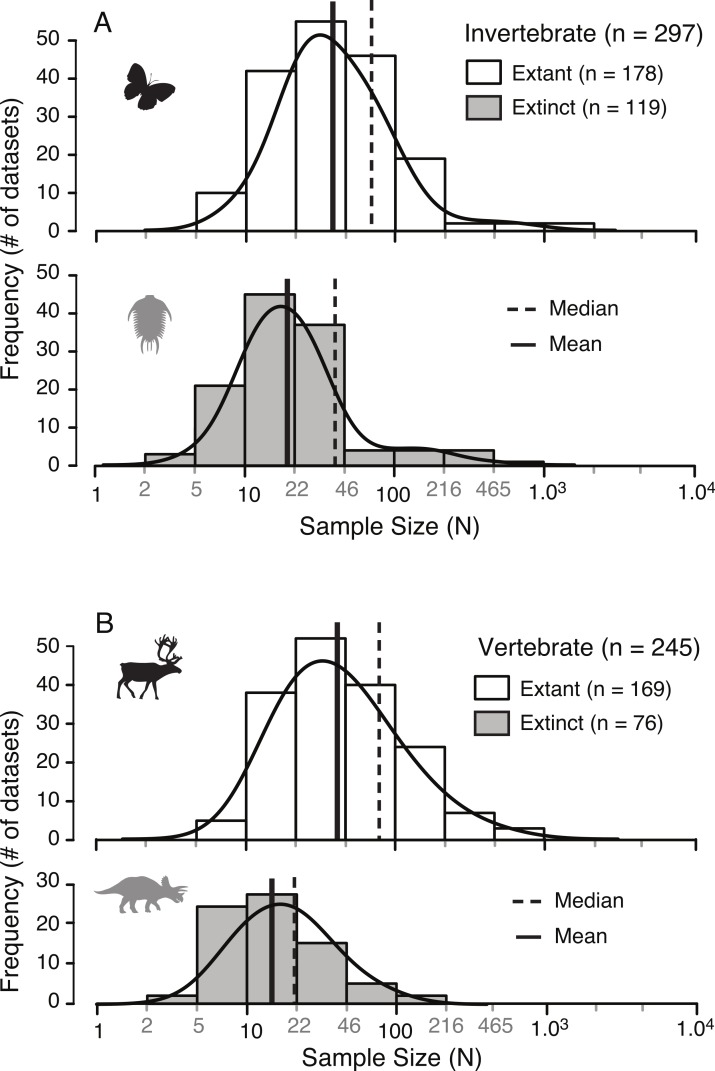
Distribution of sample sizes in published studies of intraspecies allometry. Distribution of 542 sample sizes in published studies examining intraspecies allometry, or ontogenetic trajectories, in invertebrates (A) and vertebrates (B). Solid vertical lines indicate the median, and dotted vertical lines indicate the mean.

**Figure 4:**
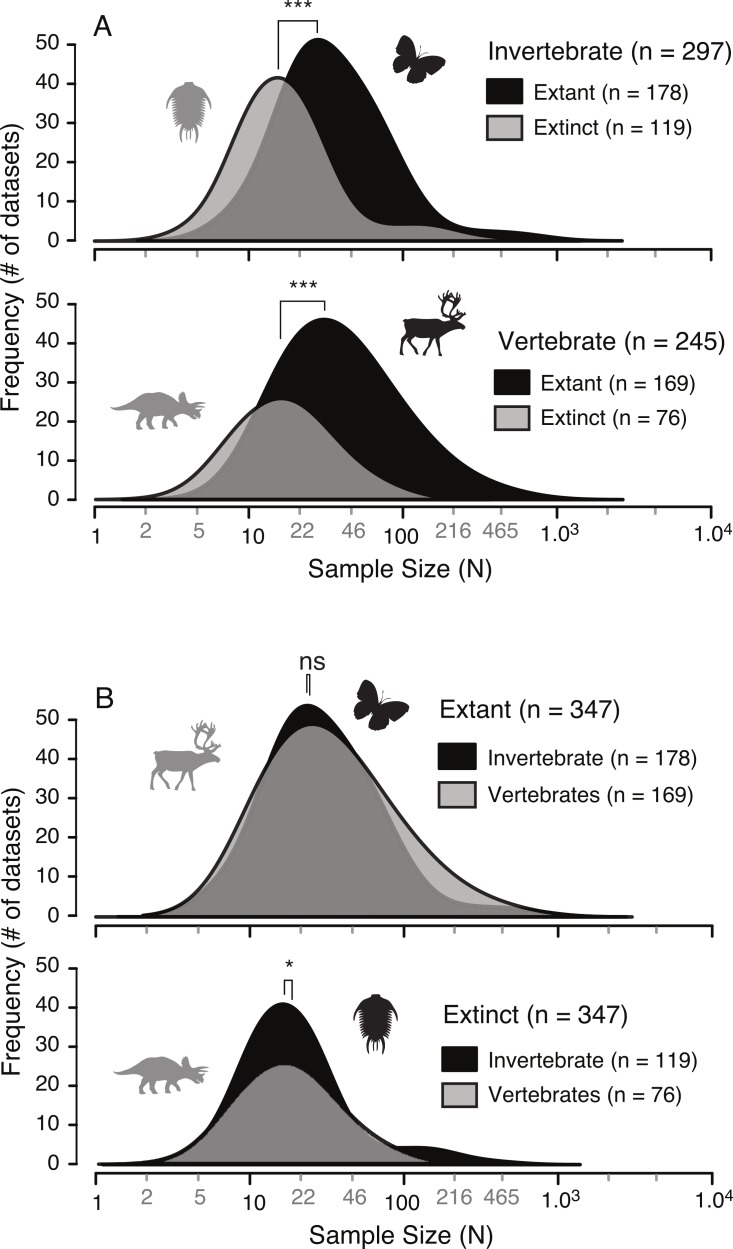
Comparison of allometric sample size distributions between extant and extinct taxa (A) and between invertebrates and vertebrates (B). Extinct taxa show a systematically smaller sample size in both invertebrates and vertebrates. Conversely, the sample sizes between invertebrates and vertebrates, for both extinct and extant taxa, are similar. “*” indicates significance of results of Kolmogorov–Smirnov tests for differences in distributions.

A survey of the literature reveals a wide range of sample sizes (*n* = 542, range = 3–1,449) used for quantifying intraspecific allometry. When these studies are segregated based on their taxa of interest (i.e., vertebrate vs. invertebrate) and age/nature (i.e., extant/recent vs. extinct/fossil), distinct patterns are clear (Fig. 3 and Table 2). Samples from extant invertebrates and extant vertebrates illustrate very similar distributions, which are not significantly different from each other (KS test, *p*-value = 0.4381) (Fig. 4 and Table 3). In contrast, those studies examining extinct taxa use systematically smaller sample sizes than those of extant taxa, a pattern that is consistent for both vertebrates and invertebrates (*p*-values <0.001) (Fig. 4 and Table 3). Although not nearly as distinct as the pattern between extinct and extant (for either group), the difference between extinct vertebrate and extinct invertebrate samples is significant (*p*-value = 0.0375). The mean, median, minimum, and maximum of the extant samples (both vertebrate and invertebrate) are all larger than those of the extinct samples. The systemic use of smaller datasets for extinct taxa can be illustrated in that only 5.6% (invertebrate) and 3.0% (vertebrate) of the extant samples are based on 10 specimens or fewer, while 20.1% (invertebrate) and 34.7% (vertebrate) of the extinct samples are of this size.

**Table 2:** The sample of published sample sizes in studies examining interspecies allometry or ontogenetic trajectories in both invertebrates and vertebrates, in both extant and extinct taxa.

	N	Min	Max	Mean	Median	*n* ≤ 10
Invertebrate extant	178	6	1,449	72.3	32.5	5.6%
Vertebrate extant	169	6	984	75.7	40.0	3.0%
Invertebrate extinct	119	3	733	39.7	19.0	20.1%
Vertebrate extinct	76	4	110	21.4	14.0	34.7%

**Table 3:** The results of a Kolmogorov–Smirnov test of the allometric study sample sizes, testing the hypothesis that the two samples were drawn from the same distribution.

	*p* value	Significance
Invertebrate: Extant vs. Extinct	1.74E–10	***
Vertebrate: Extant vs. Extinct	2.89E–10	***
Extant: Vertebrate vs. Invertebrate	0.4381	
Extinct: Vertebrate vs. Invertebrate	0.03757	*

### Minimum sample size for determination of allometry

The minimum sample size for which 95% of the replicates result in the same scaling trend as that of the entire sample are listed in Table 4. This includes the results for all subsampling methods, and for both OLS and RMA. The minimum sample required is only listed for results of allometric trends (positive or negative), and not listed for results of isometry, as it is the null hypothesis.

**Table 4:** Results of allometric analysis for OLS and RMA regression of the 22 cranial skull variables in *A. mississippiensis*. For each variables, the *R*^2^, intercept, slope, lower 95% confidence interval for the slope, upper 95% confidence interval for the slope, scaling category, significance of scaling trend, and the minimum sample size required for 95% of replicates to return a result of allometry.

								Min. Sample for 95% Allo.			
OLS Var.	*R* ^2^	Intercept	Slope	lCI	uCI	Trend	Sig.	Rand.	Occ.	Leng.	Adult
23	0.928	−0.408	0.651	0.616	0.685	–	****	7	6	7	16
10	0.969	−0.073	0.666	0.643	0.689	–	****	6	5	4	18
9	0.981	−0.103	0.737	0.717	0.757	–	****	6	5	4	15
15	0.951	−0.541	0.784	0.749	0.818	–	****	15	12	>10	>20
13	0.974	−0.860	0.916	0.887	0.944	–	****	43	>20	>10	>20
5	0.990	−0.335	0.946	0.922	0.969	–	***	61	>20	>10	>20
22	0.977	−0.571	0.959	0.931	0.988	–	**	98	>20	>10	>20
12	0.976	−0.986	0.969	0.940	0.998	–	*	>100	>20	>10	>20
14	0.993	−0.244	0.991	0.974	1.007	h. iso	0.2266	>100	>20	>10	>20
17	0.988	−0.445	0.996	0.975	1.017	h. iso	0.7146	>100	>20	>10	>20
3	0.993	−0.379	1.003	0.987	1.020	h. iso	0.6929	>100	>20	>10	>20
8	0.994	−0.567	1.010	0.994	1.025	s. iso	0.1199	>100	>20	>10	>20
4	0.990	−0.327	1.017	0.997	1.038	s. iso	0.0898	>100	>20	>10	>20
2	0.994	−0.491	1.025	1.010	1.040	+	**	77	>20	>10	>20
19	0.986	−0.746	1.033	1.009	1.057	+	**	95	>20	>10	>20
11	0.977	−1.247	1.043	1.012	1.073	+	**	95	>20	>10	>20
1	0.991	−0.687	1.052	1.032	1.072	+	****	36	>20	>10	>20
16	0.981	−0.702	1.054	1.026	1.082	+	***	64	>20	>10	>20
20	0.982	−1.218	1.066	1.038	1.093	+	****	53	>20	>10	>20
18	0.991	−0.970	1.089	1.070	1.109	+	****	18	15	8	>20
21	0.984	−1.133	1.100	1.073	1.127	+	****	35	>20	>10	>20
7	0.995	−0.562	1.132	1.117	1.147	+	****	12	9	7	>20

								Min. Sample for 95% Allo.			
RMA Var.	*R* ^2^	Intercept	Slope	lCI	uCI	Trend	SIG	Rand.	Occ.	Leng.	Adult
23	0.928	−0.465	0.675	0.641	0.711	–	****	8	7	7	>20
10	0.969	−0.098	0.677	0.654	0.700	–	****	7	6	4	>20
9	0.981	−0.120	0.744	0.724	0.765	–	****	8	6	4	>20
15	0.951	−0.587	0.804	0.770	0.839	–	****	18	15	>10	>20
13	0.974	−0.888	0.928	0.899	0.957	–	****	54	>20	>10	>20
5	0.990	−0.354	0.954	0.930	0.978	–	***	76	>20	>10	>20
22	0.977	−0.597	0.971	0.943	1.000	–	*	>100	>20	>10	>20
12	0.976	−1.013	0.981	0.952	1.010	h. iso	0.1918	>100	>20	>10	>20
14	0.993	−0.253	0.994	0.978	1.011	h. iso	0.4874	>100	>20	>10	>20
17	0.988	−0.459	1.002	0.981	1.023	h. iso	0.8428	>100	>20	>10	>20
3	0.993	−0.387	1.007	0.991	1.023	h. iso	0.4070	>100	>20	>10	>20
8	0.994	−0.574	1.013	0.998	1.029	s. iso	0.0983	>100	>20	>10	>20
4	0.990	−0.340	1.023	1.003	1.043	+	*	>100	>20	>10	>20
2	0.994	−0.498	1.028	1.013	1.043	+	***	67	>20	>10	>20
19	0.986	−0.763	1.041	1.017	1.065	+	***	80	>20	>10	>20
11	0.977	−1.275	1.055	1.025	1.086	+	***	75	>20	>10	>20
1	0.991	−0.698	1.057	1.038	1.077	+	****	31	>20	>10	>20
16	0.981	−0.725	1.064	1.036	1.092	+	****	48	>20	>10	>20
20	0.982	−1.240	1.075	1.048	1.103	+	****	42	>20	>10	>20
18	0.991	−0.981	1.094	1.074	1.114	+	****	15	13	8	>20
21	0.984	−1.154	1.109	1.083	1.136	+	****	29	>20	>10	>20
7	0.995	−0.568	1.134	1.120	1.149	+	****	11	8	7	>20

**Notes.**

lCI lower confidence interval uCI upper confidence interval Rand. Random subsample Occ. Occupancy binned subsample Leng. Length binned subsample Adult Adult biased subsample Allo. Allometry Iso. Isometry h. iso. Hard isometry s. iso. Soft Isometry

For the random subsampling method, the sample size at which 95% of the replicates result in the same scaling trend (i.e., positive/negative allometry, isometry) as that of the entire sample ranged from 6 to 95 specimens for OLS and from 7 to 80 specimens in RMA (Table 4). For both OLS and RMA, five of the variables were found to be isometric at 100 specimens, and these may require samples of greater than 100 specimens to determine a subtle allometric trend, or may continue to represent isometry with further sampling.

Due to the low range of sample sizes over which subsampling could be performed for the ‘Even Occupancy Binned,’ ‘Equal Length Binned’ and ‘Adult Biased’ methods, few variables were able to be identified as allometric (Table 4), and those that were represent extreme allometry. For the variables that were found to be allometric under these alternative subsampling methods, those that spread out the selected specimens maximally across the size range (‘Even Occupancy Binned’ and ‘Even Length Binned’) reduced the sample size needed for a correct conclusion of allometry relative to the random subsampling. The reduction of the number of specimens needed ranges from 1 to 3 (73–88% of the sample size of random) for ‘Even Occupancy Binned,’ and from 0 to 10 (44–100% of the sample size of random) for ‘Even Length Binned’ for both OLS and RMA.

Conversely, methods that attempted to simulate more realistic sampling (i.e., disproportionate sampling of certain size classes—in this case ‘Adult Biased’), resulted in a systematic increase in the sample size needed for a correct conclusion of allometry relative to the random sample. This increase in required sample size ranges from 9 to 12 (130%–200% of the sample size of random). These results highlight that it is not merely the number a specimens in the sample that affects the ability to identify allometric trends, but that perhaps equally important is the range and distribution that these samples occupy across the size range. It should also be noted that it is not always possible to determine the potential size range for a given taxa, and that missing data from the extreme endpoints will likely have a disproportionally high effect on the scaling analyses.

### Prominence of scaling trends due to sample size

**Figure 5:**
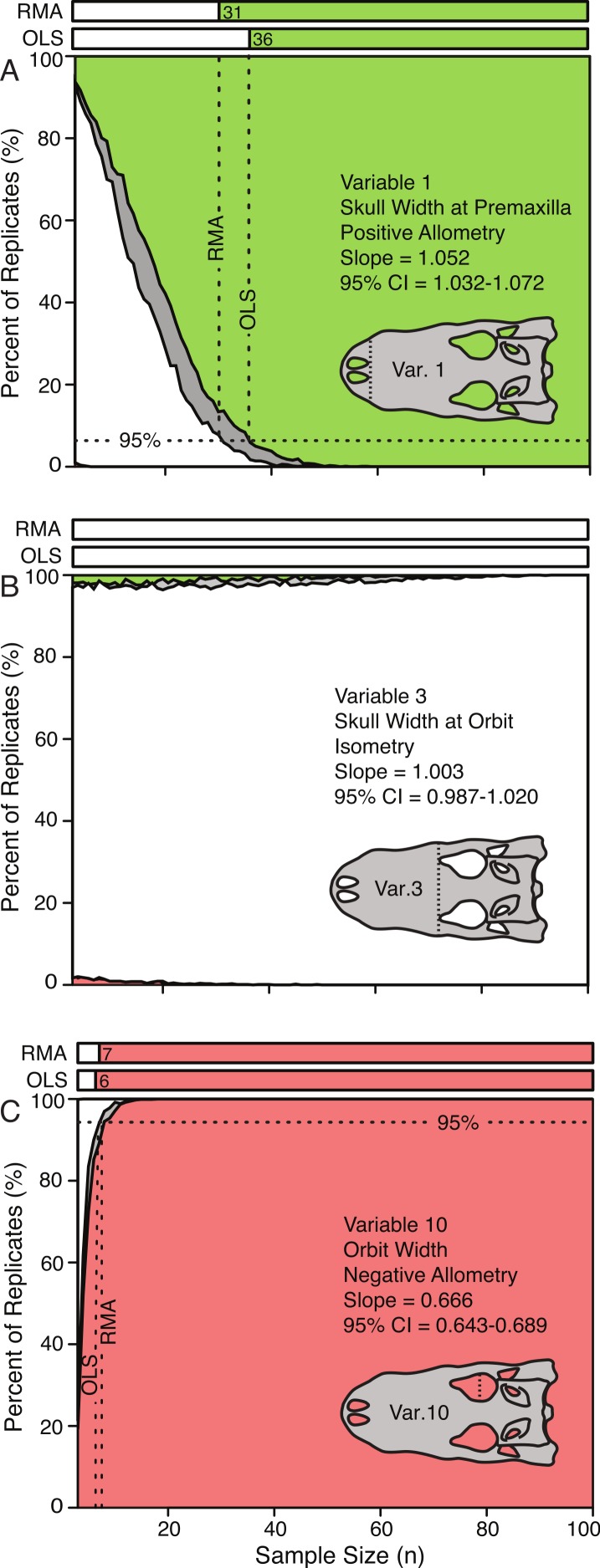
Allometric power plots illustrating the effect of sample size (random subsample) on the scaling trend of three representative variables. Variable 1, positively allometric (A); Variable 3, isometric (B); Variable 10, strongly negatively allometric (C). In all cases, white indicates isometry, green indicates positive allometry, red represents negative allometry, and grey indicated disagreement between OLS and RMA. The bars at the top represent the minimum sample size needed to achieve the same scaling trend as the entire dataset. For all variables see Fig. S2.

The effect of sample size on the conclusions of scaling trends can be visualized through the use of allometric power plots (Fig. 5 and Fig. S2), which plot the proportion of subsample replicates resulting in the categorical results (i.e., positive allometry, isometry, and negative allometry) against sample size. For all variables, subsampling methods, and regression types, the trends at low sample sizes were dominantly isometry, and as the sample size increased the percentage of replicates that were isometric either decreased (for ‘true’ allometry) or increased (for ‘true’ isometry). The sample size at which 95% of the replicates had the same result as the ‘true’ trend (i.e., that of the entire sample), for which the true trend was allometric was determined and recorded. This represents the sample size required for 95% confidence in a conclusion of allometry for that particular variable. When the ‘true’ trend was isometric, however, the level at which 95% of the replicates resulted in the correct trend was more difficult to determine, as the smallest samples usually resulted in the correct conclusion.

### Correlation of slope and minimum number of specimens

**Figure 6:**
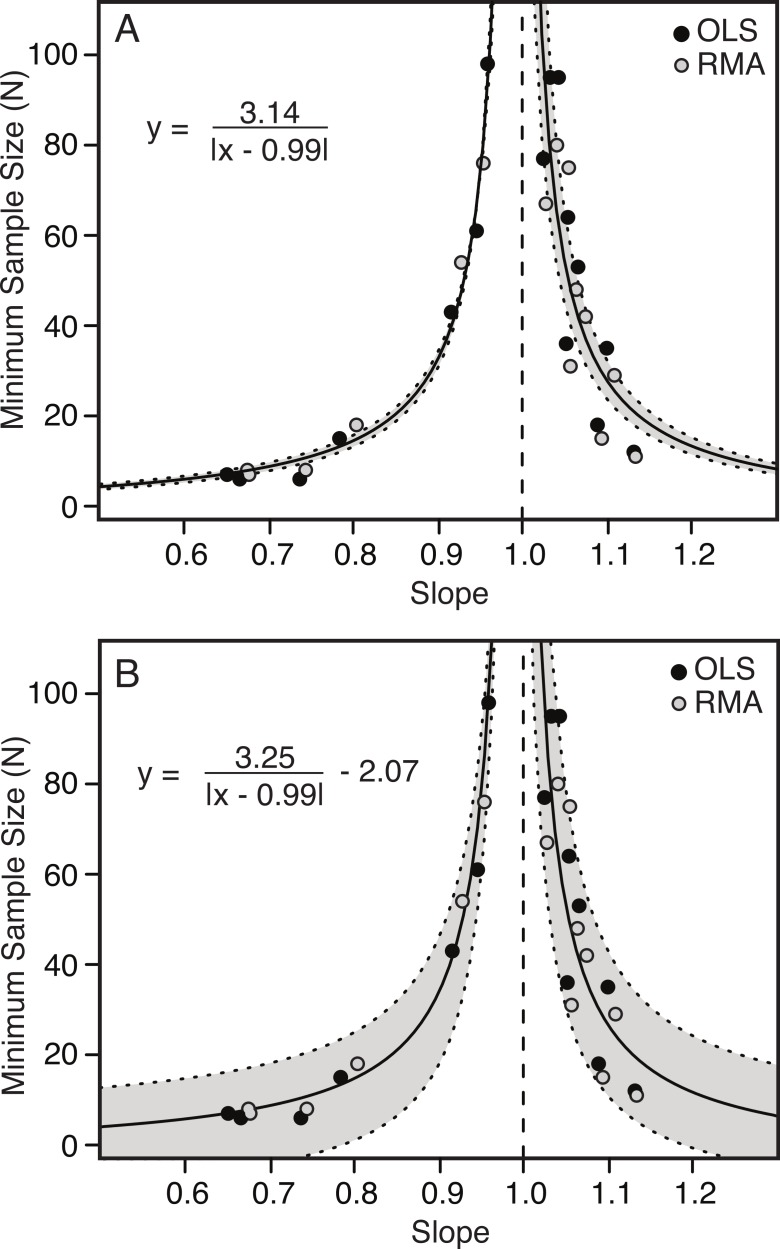
Minimum sample size required for correct identification of scaling category (in 95% of replicates) as a function of slope. Each point represents one of the 22 variables found to be allometric (with the required sample size plotted against the slope) for OLS (black) and RMA (grey). Minimum required sample sizes are small away from 1.00 (i.e., strongly allometric) and increase exponentially to vertical asymptotes as the slope approaches 1.00 (i.e., isometric). The relationship is best described by a hyperbolic function (solid line), with 95% confidence intervals indicated in grey. The fitted model includes both the RMA and OLS data. Simpler model (Eq. (1.1)) with two parameters (A). More complex model (Eq. (1.2)) with third term, allowing for error in *y*-axis (B). The vertical dashed line indicates a slope of 1.00.

There is a strong correlation between the slope of the relationship between two variables and the minimum number of specimens needed to determine a scaling category with 95% confidence. The further the slope deviates from 1.00 (either positively or negatively) the fewer specimens are needed to conclude allometry (Table 4 and Fig. 6). Conversely, as the slope becomes closer to 1.00, the number of specimens increases dramatically.

The relationship between slope and minimum sample size is inverse and hyperbolic, with vertical asymptotes at a slope of just less than and just greater than 1.00, and horizontal asymptotes at a minimum sample size for slopes deviating greatly from 1.00 (Fig. 6). The basic equation describing the relationship is shown by Eq. (1.1), where ‘*m*’ describes the shape of the curve and ‘*b*’ describes the position of the vertical axis (Fig. 6A). An additional term ‘*c*’ can be added to this equation (see Eq. (1.2)), which allows for additional error in the *y*-axis (Fig. 6B)

(1.1) \begin{eqnarray*} y=\frac{m}{\vert x-b\vert } \end{eqnarray*}

(1.2) \begin{eqnarray*} y=\frac{m}{\vert x-b\vert }+c. \end{eqnarray*}

The results comparing the goodness of fit of the two equations, penalizing complexity using Akaike Information Criterion (AIC) are shown in Table 5. In all cases Eq. (1.1) is preferred to Eq. (1.2), but this difference is marginal.

**Table 5:** Results of model fitting of Eqs. (1.1) and (1.2) to the empirical data for the minimum number of specimens required for determination for allometry (95% confidence) in the crocodilian skull variables. Results include those for both combined and separate OLS and RMA regressions.

	Combined RMA/OLS		RMA		OLS	
	Eq. (1.1)	Eq. (1.2)	Eq. (1.1)	Eq. (1.2)	Eq. (1.1)	Eq. (1.2)
Residual SS	4,925	4,889	2,773	2,740	2,077	2,075
AIC	251.087	252.855	133.885	135.698	122.531	124.512
delta AIC	0.000	1.767	0.000	1.183	0.000	1.981
Akaike Weight	0.708	0.292	0.712	0.288	0.729	0.271
*m*	3.14	3.25	3.21	3.35	3.04	3.09
95% CI of *m*	(2.83–3.45)	(2.66–3.84)	(2.74–3.68)	(2.46–4.24)	(2.53–3.54)	(2.10–4.07)
*b*	0.992	0.992	0.993	0.993	0.992	0.992
95% CI of b	(0.988–0.997)	(0.988–0.996)	(0.987–0.999)	(0.987–0.999)	(0.985–0.999)	(0.984–1.000)
*c*	NA	−2.07	NA	−2.68	NA	−0.81
95% CI of c	NA	(−11.29–7.16)	NA	(−17.51–12.15)	NA	(−15.08–13.48)

### Error rate as a function of sample size

**Figure 7:**
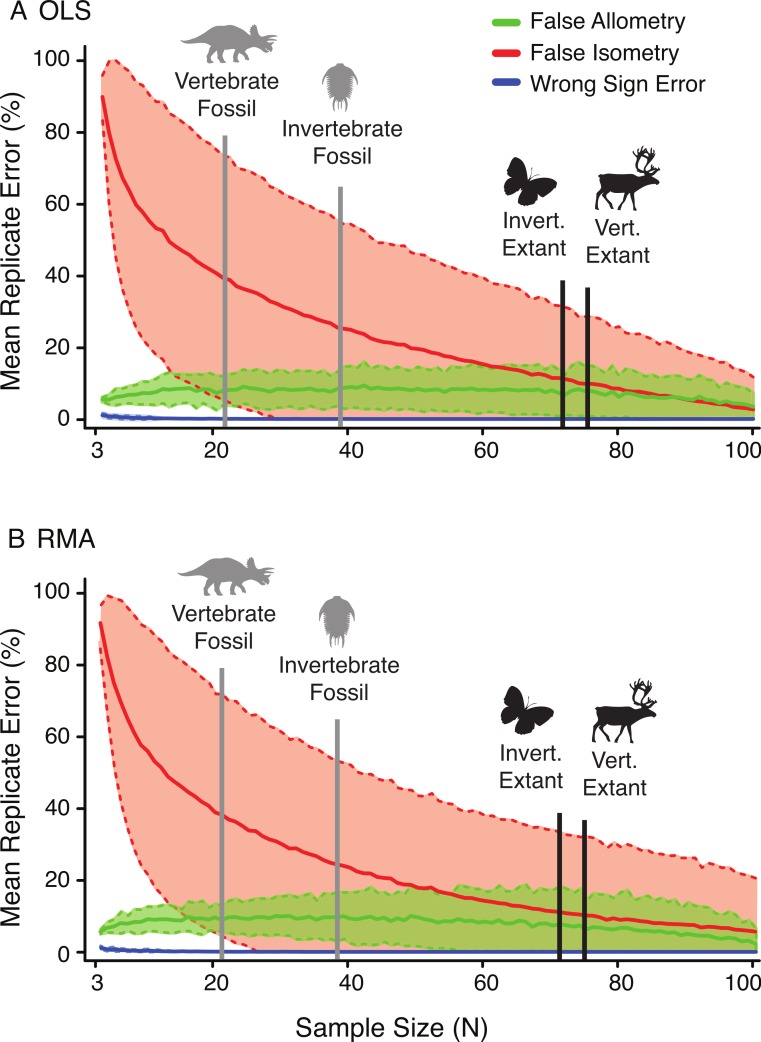
The effect of sample size on the frequency of false allometry (‘Type I error’) (green), false isometry (‘Type II error’) (red), and wrong sign error (blue) in the random subsample replicates of *A. mississippiensis* for OLS (A) and RMA (B). Solid lines represent the mean of all 22 variable replicates and dotted lines represent one standard deviation of all 22 variable replicates (derived from the *Alligator* subsampling). For relative comparison, the mean sample sizes for the literature review of allometric studies of fossil and extant, (and vertebrae and invertebrate) allometric studies are indicated with the vertical bars.

The relative rates of false allometry, false isometry, and sign error change drastically as a function of the sample size. Figure 7 illustrates the relative dominance of these errors as the sample size increases, for both OLS (A) and RMA (B) in the random subsample. In both cases, the false isometry (‘Type II error’) rate is consistently very high (mean >50% when *n* < 12) for small samples, and decreases as the sample size increases. In contrast, false allometry (‘Type I error’) and sign error rates are low, and very low respectively, (mean = ∼10% when *n* < 12, and mean <3% when *n* > 12), with false allometry rate changing little in response to increased sample size, and sign error not being a factor at sample sizes greater than twenty.

## Discussion

### Extinct and extant sample sizes

The results presented here illustrate that, unsurprisingly, studies of intraspecific ontogenetic allometry based on extinct animals consistently use smaller sample sizes than those based on living animals (Fig. 4). Similar disparity between sample sizes of extinct and extant taxa in both vertebrates and invertebrates, and the similarity between sample sizes of both extant vertebrates and invertebrates and, to a lesser extent, extinct vertebrates and invertebrates suggest these small sample sizes are a result of work on extinct taxa, irrespective of whether they are vertebrate or invertebrate. This distinction between sample sizes of extant and extinct taxa is driven by the nature of the specimens available, namely that investigations into allometry in extinct taxa require fossil datasets. In this regard, palaeobiologists are restricted to using those data preserved in fossil record, which greatly restricts the number and types of specimens available.

In addition to their rarity, the collection of fossil specimens is often more difficult than collection of their extant counterparts. Fossil samples often require great investments of time and money for prospecting, excavation, and preparation and often represent a finite supply that is soon exhausted. This also acts to limit the number of specimens available. When large numbers of specimens from one species can be obtained, these samples often see two distinct and negative effects of taphonomic processes. Firstly, many of the specimens may suffer from either incompleteness or distortion, making them difficult or impossible to use in allometric studies (Brown, Arbour & Jackson, 2012; Strauss & Atanassov, 2006). Secondly, taphonomic biases often act on the absolute size of the organism, and as a result may skew the relative abundance of samples across the ontogenetic trajectory (e.g., biases reducing the abundance of small-bodied specimens) (Behrensmeyer, Western & Dechant Boaz, 1979; Brown et al., 2013; Kidwell & Flessa, 1996). Occasionally, large samples of vertebrate fossils suitable for allometry studies can be recovered, but in many of these cases they are often restricted to small portions of the anatomy that are both taphonomically resistant and taxonomically informative (e.g., teeth, pachycephalosaur domes) (e.g., Evans et al., 2013). Despite these limitations, the fossil record offers unique data not available in neontological datasets, specifically for studies of evolutionary biology requiring deep time data.

Although this study largely focuses on small sample size as a limiting factor for scaling analyses based on extinct taxa, this phenomenon is by no means restricted to the fossil record. Specimen collection of certain extant taxa may be more difficult than in well-represented fossil taxa. Studies investigating scaling in extant taxa that are rare, critically endangered, not normally part of museum collections, exotic, or restricted to inaccessible areas will face similar limitations. Rather than having implications for scaling in fossil species only, this study has relevance to scaling analysis in any system where obtaining a large (or well-distributed) sample is difficult.

### False allometry, false isometry and sample size

Given the results from the empirical *A. mississippiensis* data, it is likely impractical to delimit a generally applicable distinct minimum sample size that is recommended for allometry studies. When the sample size is low, the statistical power is reduced, and the confidence intervals become wider, making the null hypothesis more difficult to reject (high Type II error rate at low sample size) (Fig. 7). This trend is consistent across all variables, regression types, and subsampling methods. Inferences of allometry are relatively robust regardless of sample size. In contrast, inferences of isometry are affected by high Type II error at low sample sizes, and may therefore indicate less about the relative growth of a structure and more about the statistical power of the analysis (Fig. 7).

The sample size at which high false isometry (‘Type II error’) occurs is dependent upon the slope of the variable, but the range of sample size for the 22 cranial characters of *A. mississippiensis* is illustrated in Fig. 7. The mean of false isometry rate is consistently higher than the mean false allometry for all sample sizes smaller than ∼70 specimens for both OLS and RMA. Above ∼70 specimens the rates of false isometry and false allometry are relatively similar. Although the general pattern seen here should be consistent across taxa of varying phylogenic histories and scales, it is unclear how well this specific threshold will predict scaling limitations in other taxa. Further investigations on other taxa will help to determine how representative the empirical data for *A. mississippiensis* are for other taxa and other systems.

Importantly, the mean sample for both fossil invertebrates (39.7) and vertebrates (21.4), derived from the literature review, are well below the high false isometry threshold in *Alligator*, while those of extant invertebrates (72.3) and vertebrates (75.7) are at or above this region of equal error rate (Fig. 7). This suggests that the majority of analyses based on fossil data may suffer from significant amounts of Type II error, resulting in disproportionate levels of isometry. This is not the case for the majority of studies based on extant species. This could lead to the misleading result that isometry is, on average, more common in extinct taxa, due to smaller sample sizes. Whether the prevalence of isometry is significantly higher in studies of fossil taxa relative to recent taxa is currently unknown, but this represents a logical prediction as long as the samples size patterns are consistent with those observed here.

### Allometric nomenclature and the false dominance of isometry

For ‘true’ isometry the rate of change of one variable relative to another is exactly 1.00 (when the variables under comparison have the same dimensionality). This is only one of the infinite number of possible slopes between the two variables, with all other possible slopes being allometric (either positive or negative). Given this, combined with factors such as measurement error and significant figures (Simpson, Roe & Lewontin, 1960) a slope of exactly 1.00 should be rare in biological datasets, but in many cases it is seen as the default. In the context of a model of geometric similarity, isometry is the null hypothesis, failing to reject a test for isometry does not require a slope of 1.00, just that the slope is not significantly different than 1.00, which will occur with small sample sizes. It is important to note that this is not necessarily the case under models of elastic or static stress similarity where these two models present alternative hypotheses (Biewener, 2005; McMahon, 1975). Interestingly, similar implications to those discussed here may occur in non-morphological traits that scale with body size, such as physiological traits (e.g., metabolic rate) and/or ecological traits (e.g., range size). Further research on these systems will determine how broadly applicable the result of this analysis are.

**Figure 8:**
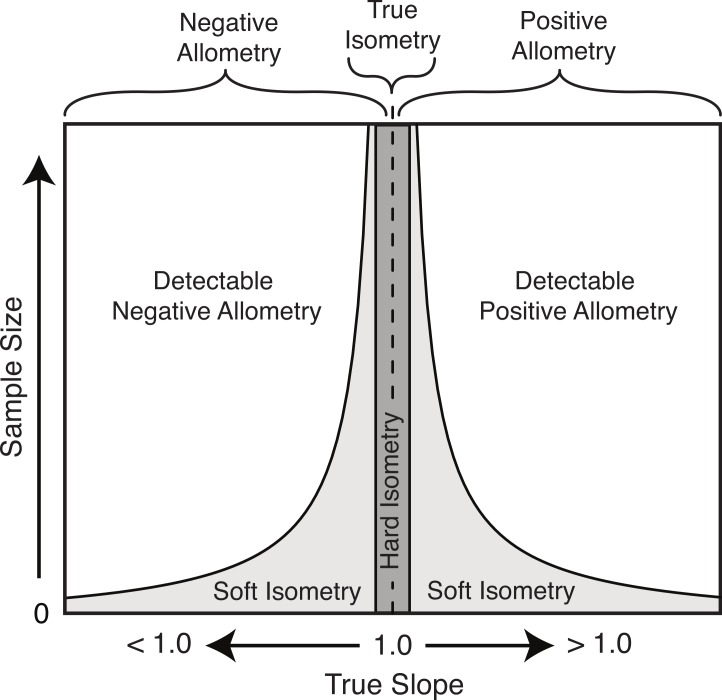
Schematic of the relationship of the true slope and the sample size to the ability to categorize scaling trends.

Based on the results herein, we suggest a modification to the nomenclature of isometry to clarify this potential imprecise terminology. The term ‘true isometry’ is suggested for the case of the slope being equal to exactly 1.00. This is largely a theoretical concept in the context of biology, and would be impossible to prove with empirical data. The term ‘hard isometry’ is suggested for the case in which the slope is not statistically different from 1.00, and continued sampling will not change this result (i.e., the result is not due to low sample size or low power) (Fig. 8). Conversely, the term ‘soft isometry’ is suggested for the case in which the slope is not statistically different from 1.00, but this is due to low sample size and will become statistically different from 1.00 with further sampling (Fig. 8). Allometry, not being prone to high error related to sample size, does not require further subdivision.

The use of ‘hard’ and ‘soft’ to indicate the level of confidence in the isometric relationship is borrowed from the similar usage in phylogenetics (Maddison, 1989). A ‘soft’ polytomy is the situation of unresolved branching pattern due to insufficient or conflicting phylogenetic resolution. Likewise, ‘soft’ isometry is used to indicate the uncertainty in the scaling trend due to low statistical power. Conversely, a ‘hard’ polytomy is used to describe the interpretation of having multiple, simultaneous speciation events associated with a single common ancestor (interpretation of a biological phenomenon). Likewise, ‘hard’ isometry is used to indicate interpretation of equal scaling of two variables, within statistical error.

As with hard and soft polytomies, distinguishing between hard and soft isometry may be not be easy, but the distinction between them is important as they lead to different biological interpretations. Isometric results based on small samples should be interpreted as soft isometry, and due to low statistical power. Subsampling of large datasets can reveal how the isometric result changes with sample size and can allow for interpretation of hard isometry.

Alternately, rather than utilizing categorical divisions (i.e., positive allometry, negative allometry, and isometry), it may prove to be useful to report and discuss these scaling questions in the context of standardized metrics including samples size, sample range, slope, confidence intervals of slope, *R*^2^, and significance values.

## Supplemental Information

10.7717/peerj.818/supp-1 Figure S1 Visualization of the effect of measurement error on the allometric analysis Measurement error in both standard deviation (A) and mm (B) as a function of measurement magnitude (mm). Size distribution of measurement error relative to the measurements and the rounding threshold.

10.7717/peerj.818/supp-2 Figure S2 Allometric power plots illustrating the effect of sample size on the allometric trend of all variables The horizontal axis indicates subsample size and the vertical axis indicated percentage or replicates of certain allometric trend. White indicates isometry, green indicates positive allometry, red represents negative allometry, and grey indicated disagreement between OLS and RMA. The bars at the top represent the minimum sample size needed to achieve the same allometric trend as the entire dataset.

10.7717/peerj.818/supp-3 Table S1 Dataset with the results of a literature survey investigating the sample sizes used in intraspecies allometric studies Each sample includes the literature reference, genus and species, whether it is invertebrate or vertebrate and extinct or extant, and the sample size (N).

10.7717/peerj.818/supp-4 Table S2 Dataset of the cranial measurements of specimens of *Alligator mississippiensis* used in this study Dataset of 108 specimens of *Alligator mississippiensis*, as well as their cranial measurements (23) used in this study. Institutional Abbreviations; AM, Australian Museum, Sydney; AMNH, America Museum of Natural History, New York; CMN, Canadian Museum of Nature, Ottawa; FMNH, Field Museum of Natural History, Chicago; TMM, Texas Memorial Museum, Austin; ROM, Royal Ontario Museum, Toronto; RTMP, Royal Tyrrell Museum of Palaeontology, Drumheller; UCMP, University of California Museum of Paleontology, Berkeley; UCMVZ, University of California Museum of Vertebrate Zoology, Berkeley; UCMZ, University of Calgary Museum of Zoology, Calgary; UF, Florida Museum of Natural History, Gainesville; UM, University of Michigan Museum of Zoology, Ann Arbour; USMN; National Museum of Natural History (Smithsonian), Washington.
