# Supplementary material for: Small sample sizes in the study of ontogenetic allometry; implications for palaeobiology
Source: PeerJ. 2015 Mar 10;3:e818. doi: 10.7717/peerj.818 (PMC4358694; doi:10.7717/peerj.818)
Supplement: Figure S2 — The horizontal axis indicates subsample size and the vertical axis indicated percentage or replicates of certain allometric trend. White indicates isometry, green indicates positive allometry, red represents negative allometry, and grey indicated disagreement between OLS and RMA. The bars at the top represent the minimum sample size needed to achieve the same allometric trend as the entire dataset. [file peerj-03-818-s002.pdf]

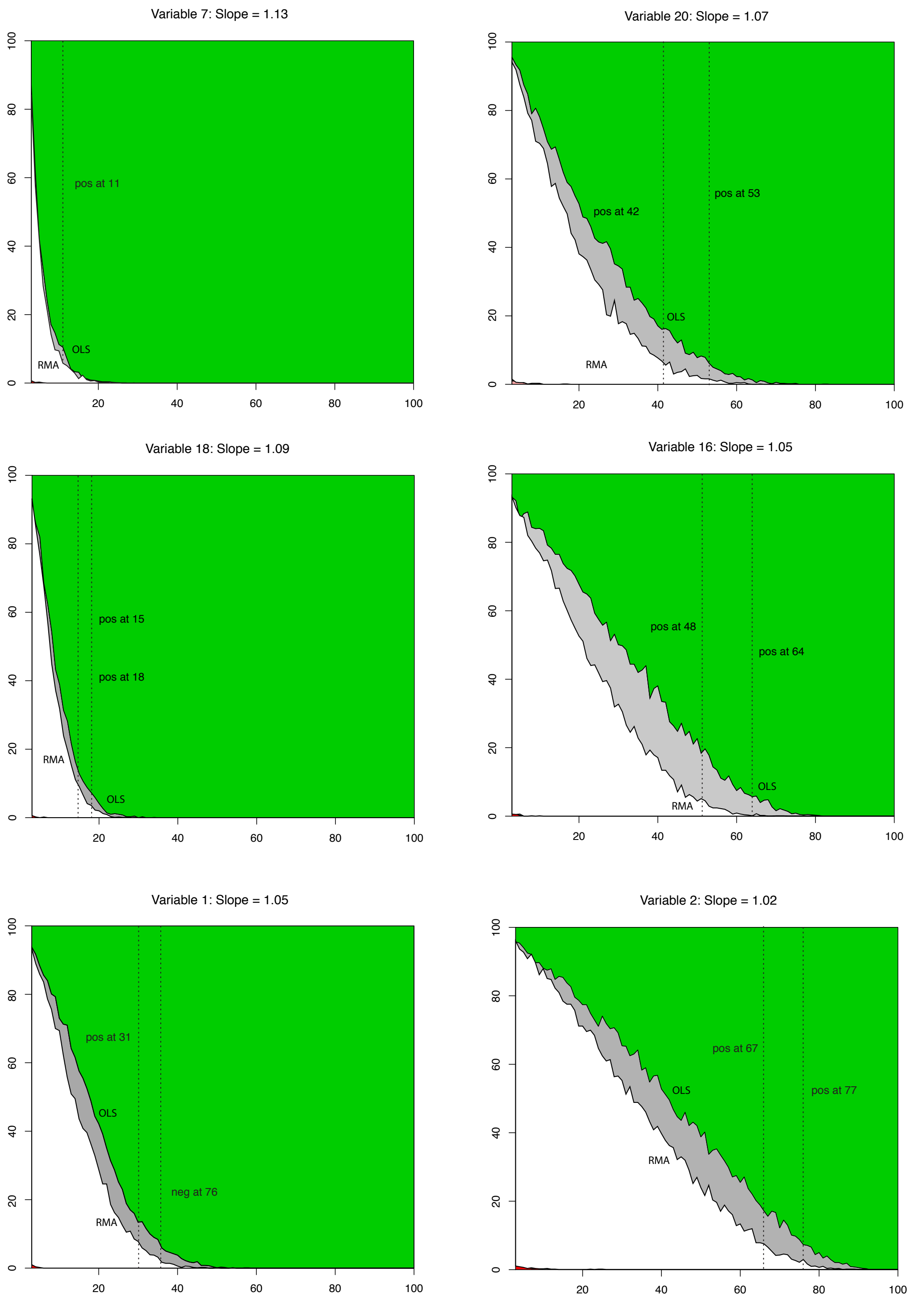

Variable 7: Slope = 1.13
pos at 11
OLS
RMA
Variable 20: Slope = 1.07
pos at 42
pos at 53
OLS
RMA
Variable 18: Slope = 1.09
pos at 15
pos at 18
RMA
OLS
Variable 16: Slope = 1.05
pos at 48
pos at 64
OLS
RMA
Variable 1: Slope = 1.05
pos at 31
OLS
neg at 76
RMA
Variable 2: Slope = 1.02
pos at 67
OLS
pos at 77
RMA
0
20
40
60
80
100

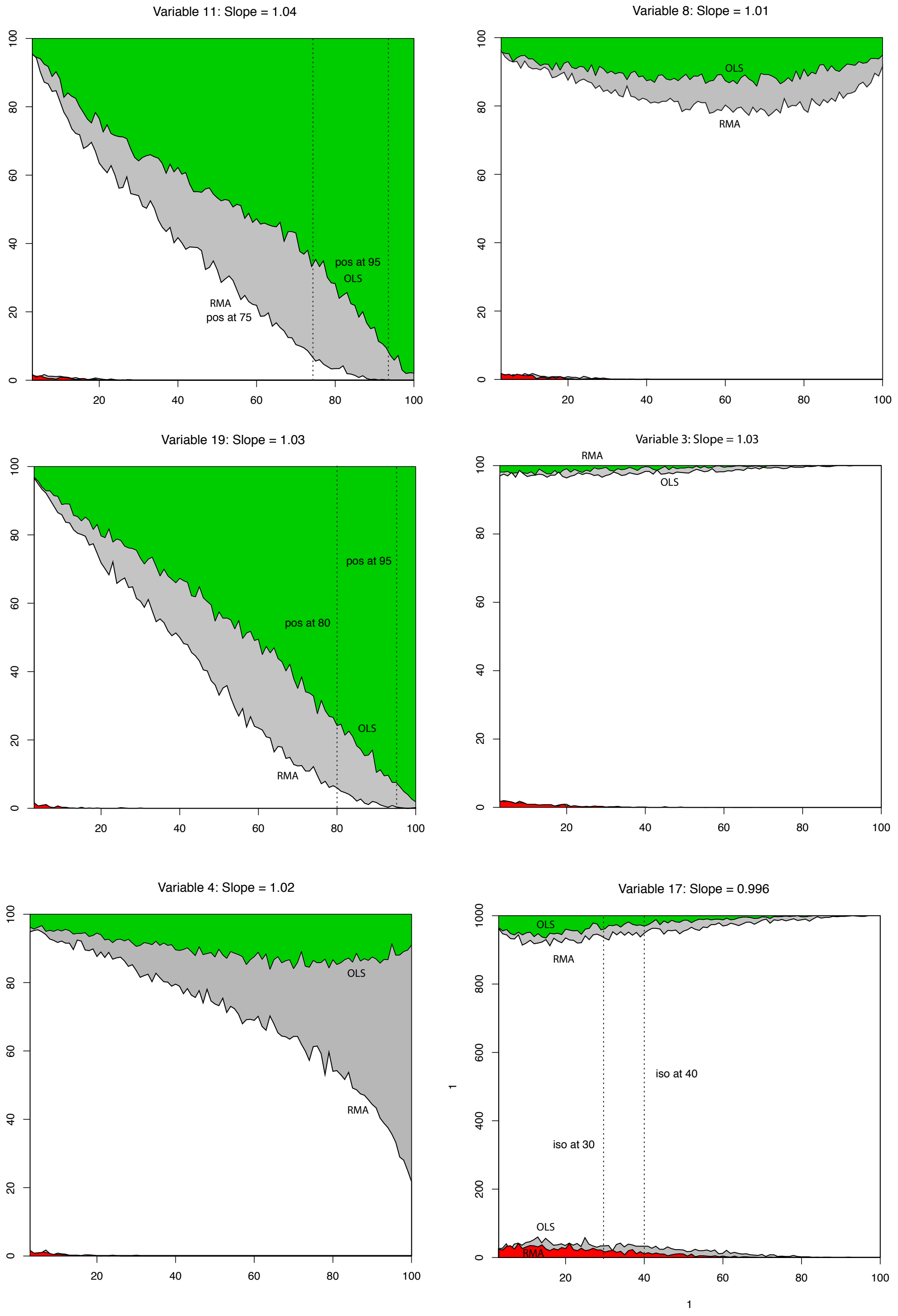
Variable 11: Slope = 1.04
pos at 95
OLS
RMA
pos at 75
Variable 8: Slope = 1.01
OLS
RMA
Variable 19: Slope = 1.03
pos at 95
pos at 80
OLS
RMA
Variable 3: Slope = 1.03
RMA
OLS
Variable 4: Slope = 1.02
OLS
RMA
Variable 17: Slope = 0.996
OLS
RMA
iso at 40
iso at 30
OLS
RMA
1
1

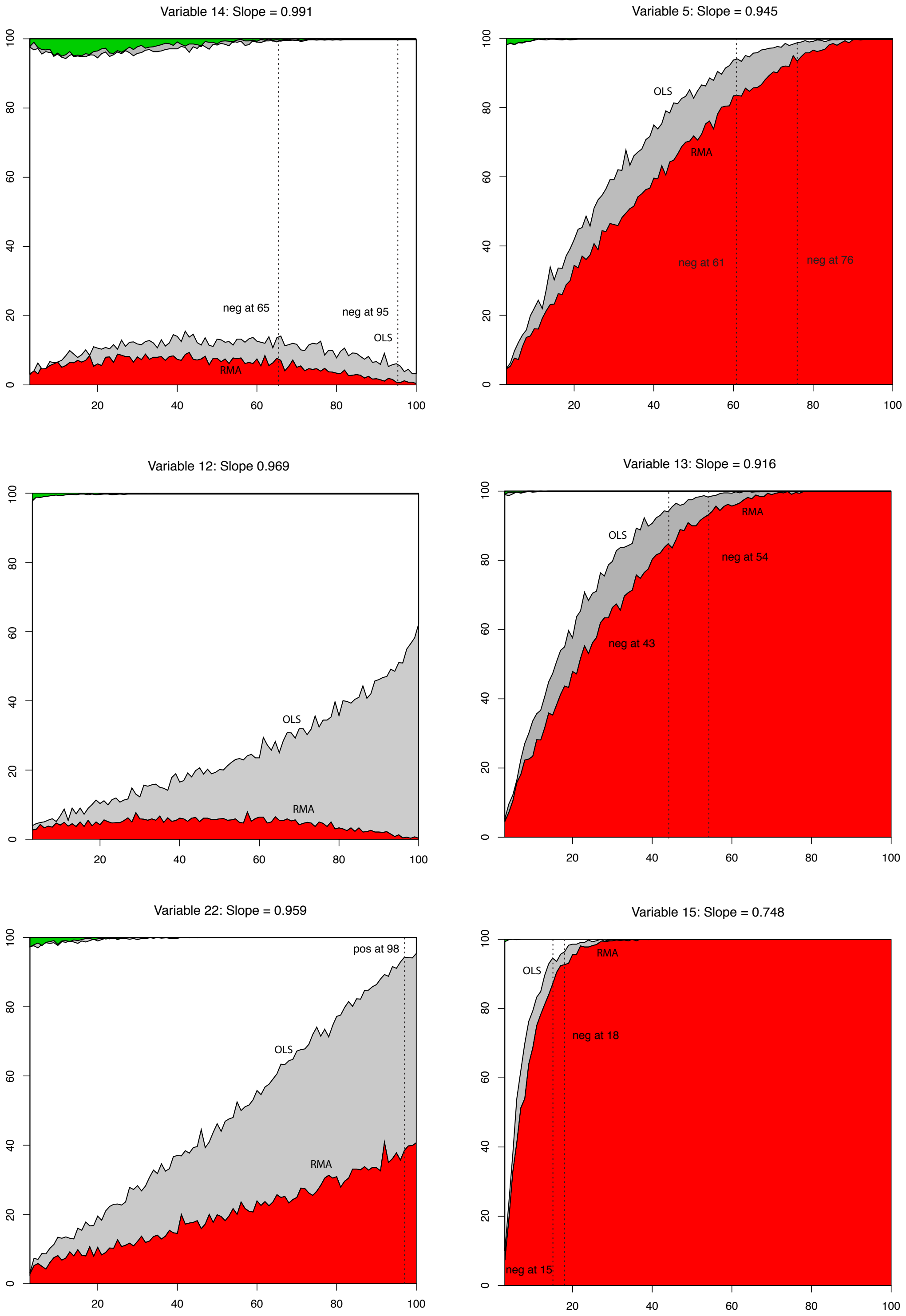
Variable 14: Slope = 0.991
neg at 65
neg at 95
OLS
RMA
0
20
40
60
80
100
20
40
60
80
100
Variable 5: Slope = 0.945
OLS
RMA
neg at 61
neg at 76
0
20
40
60
80
100
20
40
60
80
100
Variable 12: Slope 0.969
OLS
RMA
0
20
40
60
80
100
20
40
60
80
100
Variable 13: Slope = 0.916
OLS
RMA
neg at 54
neg at 43
0
20
40
60
80
100
20
40
60
80
100
Variable 22: Slope = 0.959
pos at 98
OLS
RMA
0
20
40
60
80
100
20
40
60
80
100
Variable 15: Slope = 0.748
OLS
RMA
neg at 18
neg at 15
0
20
40
60
80
100
20
40
60
80
100

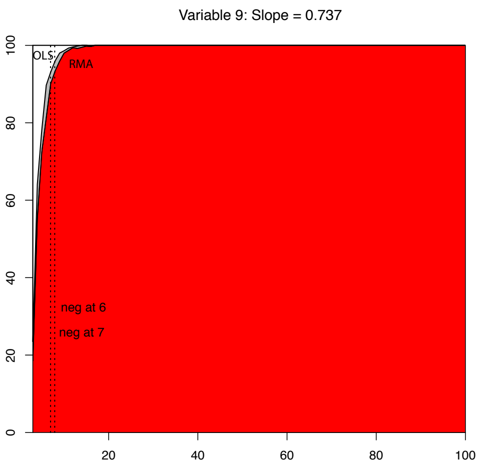
Variable 9: Slope = 0.737
OLS
RMA
neg at 6
neg at 7
0
20
40
60
80
100
20
40
60
80
100

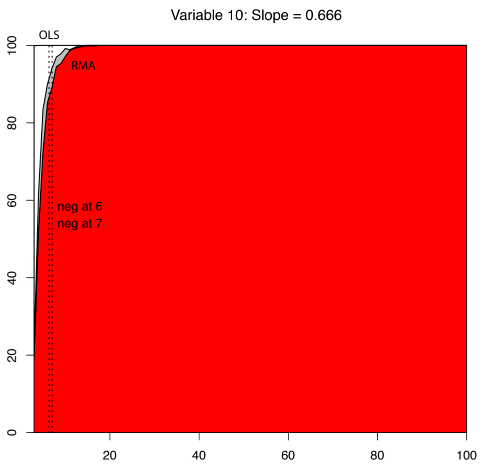
Variable 10: Slope = 0.666
OLS
RMA
neg at 6
neg at 7
0
20
40
60
80
100
20
40
60
80
100

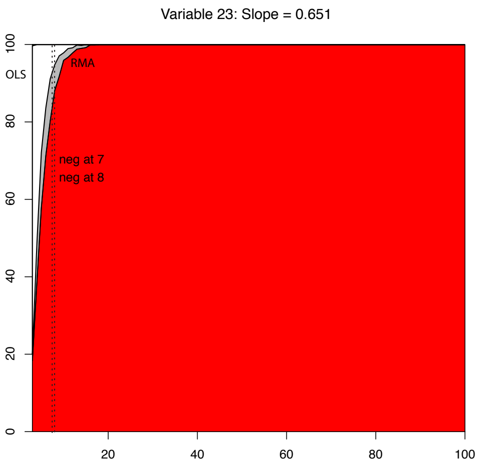
Variable 23: Slope = 0.651
OLS
RMA
neg at 7
neg at 8
0
20
40
60
80
100
20
40
60
80
100
